# Real-world pharmacovigilance of drug-induced osteoporosis: a focus on antiretroviral therapy and toxicological mechanisms in the elderly

**DOI:** 10.3389/fphar.2026.1790867

**Published:** 2026-04-07

**Authors:** Xiaoyu Liu, Yanlong Gong, Lu Liu, Xiaomin Wang, Kangyi Hu, Min Song, Yongjia Song

**Affiliations:** 1 Clinical College of Chinese Medicine, Gansu University of Chinese Medicine, Lanzhou, Gansu, China; 2 Minimally Invasive Spine Orthopedics, Affiliated Hospital of Gansu University of Chinese Medicine, Lanzhou, Gansu, China

**Keywords:** decreased bone density, genderdifferences, induction time analysis, osteoporosis, pharmacovigilance analysis, toxicological analysis

## Abstract

**Background:**

Osteoporosis and bone density reduction are significant health concerns in elderly populations. While aging is a primary factor, drug-induced bone loss—particularly associated with antiviral agents, proton pump inhibitors (PPIs), and immunomodulatory drugs—has become a growing issue in patients managing multiple conditions.

**Objective:**

This study aims to comprehensively assess adverse drug reactions (ADRs) related to bone density reduction and osteoporosis in elderly patients using the FDA Adverse Event Reporting System (FAERS). A specific focus is placed on HIV patients using antiretroviral therapy (ART), alongside an exploration of gender differences and toxicological mechanisms of high-risk drugs.

**Methods:**

Data from FAERS (2004-Q2 2025) were analyzed using disproportionality analysis (DPA) to calculate Reporting Odds Ratios (RORs) and 95% confidence intervals (CIs). Toxicological analysis was conducted on high-risk drugs (e.g., Tenofovir Disoproxil) using PubChem, GeneCards, and GO/KEGG pathway enrichment to identify disrupted biological processes.

**Results:**

Antiretroviral drugs exhibited the most significant risk signals. For decreased bone density, Emtricitabine/Tenofovir Disoproxil (ROR = 713.48, 95% CI: 648.66–784.79) and Tenofovir Disoproxil (ROR = 665.79, 95% CI: 611.91–724.42) showed extreme associations. For osteoporosis, significant signals were also identified for these antivirals, as well as for Esomeprazole (ROR = 12.23, 95% CI: 11.27–13.28) and Adalimumab (ROR = 2.16, 95% CI: 1.99–2.35). Gender-specific differences indicated men are at higher risk from antiviral drugs, whereas women are more affected by bone metabolic and immunomodulatory regulators. Toxicological analyses suggest these drugs disrupt vitamin D metabolism, calcium homeostasis, and parathyroid hormone (PTH) signaling.

**Conclusion:**

Long-term use of antiretroviral drugs, PPIs, and immunomodulators is strongly linked to bone metabolic disorders in the elderly. Although pharmacovigilance studies utilizing FAERS are limited by spontaneous reporting bias and the inability to establish direct causality, these quantitative findings and toxicological insights provide robust real-world evidence for enhancing clinical monitoring and personalized risk management.

## 1 Introduction

Decreased bone density and osteoporosis are among the most common bone metabolic diseases in the elderly, characterized primarily by bone mass reduction, trabecular structure degradation, and increased bone fragility, leading to a significant rise in fracture risk (Isganaitis, 2019). These conditions not only impair the patient’s quality of life but also cause chronic pain, decreased mobility, and long-term disability risks. In clinical practice, osteoporosis is often regarded as a natural consequence of aging. However, recent studies have shown that drug-induced bone density loss in the elderly population is also of significant clinical concern (Di et al., 2025). If drug-induced bone metabolic disorders are not promptly recognized and intervened, they may exacerbate bone loss, increase fracture risk, and lead to treatment interruptions and escalating medical costs, posing a potential threat to elderly patients’ health management and drug safety.

In long-term medication therapy for elderly patients, several commonly used drugs have been found to be associated with decreased bone density and osteoporosis. Antiviral medications, such as Tenofovir disoproxil and its fixed-dose combinations, have been shown to reduce bone mineral content and increase fracture risk (Dumitrescu et al., 2025). Proton pump inhibitors (PPIs), such as Esomeprazole, interfere with calcium absorption and bone remodeling, thereby increasing the risk of osteoporosis and hip fractures (Lespessailles and Toumi, 2022). Moreover, antitumor and immunomodulatory drugs, such as Adalimumab, while improving symptoms of inflammatory diseases, may indirectly affect bone metabolism by inhibiting osteoblast function or enhancing osteoclast activity (Ran et al., 2025). Additionally, the long-term use of aromatase inhibitors (e.g., Anastrozole, Letrozole) in postmenopausal women is closely associated with bone loss (Robertson et al., 2016). However, existing studies are mostly limited to specific drugs or single-center clinical observations, lacking systematic drug risk spectrum analyses based on real-world data.

Elderly populations exhibit significant individual differences in drug metabolism, physiological reserves, and bone metabolic responses. With age, factors such as renal function decline, decreased endocrine levels, and calcium-phosphate metabolism disorders may amplify the effects of medications on bone metabolism (Irsik et al., 2021). At the same time, the presence of multiple chronic diseases and polypharmacy complicates drug interactions, increasing the probability of adverse bone metabolic reactions (Almodovar and Nahata, 2019). However, systematic research on drug-related bone density loss and osteoporosis risk characteristics in the elderly population is still limited, particularly in areas such as high-risk drug identification, gender differences analysis, and the patterns of drug-induced time variation. This lack of quantitative evidence restricts the clinical individualized medication risk prevention and control in elderly patients.

In recent years, the osteoporosis risk in HIV patients has also gained attention. The relationship between HIV infection and decreased bone density has been confirmed in multiple studies, especially among patients using antiretroviral therapy (ART) for extended periods. These medications can significantly impact bone metabolism, leading to an increased incidence of osteoporosis (Biver, 2022).

Based on this, the present study relies on the U.S. Food and Drug Administration (FDA) Adverse Event Reporting System (FAERS) database to systematically analyze adverse event reports related to drug-induced bone density loss and osteoporosis in the elderly population from Q1 2004 to Q2 2025 (Yu et al., 2021). The study employs disproportionate analysis methods, such as reporting odds ratios (ROR), to identify potential high-risk drugs, with gender subgroup comparisons to evaluate signal differences across different groups. A potential toxicological analysis of high-risk drugs in HIV patients is also conducted. Finally, external validation is performed using the Canadian CVARDD database to ensure the robustness and credibility of the results (Thiruchelvam et al., 2025). This study aims to systematically reveal the risk spectrum, gender differences, and temporal characteristics of drug-related bone density loss and osteoporosis in the elderly population, providing real-world evidence for clinical drug safety management, and offering scientific reference for accurately identifying high-risk groups and optimizing medication strategies for elderly patients.

## 2 Materials and methods

### 2.1 Data source and process

The adverse event data used in this study were sourced from the FAERS database (https://fis.fda.gov/extensions/FPD-QDE-FAERS/FPD-QDE-FAERS.html). This database has been publicly available since 2004, and the data for adverse event reports from Q1 2004 to Q2 2025 were downloaded from the ASCII FAERS database.

For data downloaded from the FAERS database with the same “caseid” (report code), only the most recent report based on the date was retained, and duplicate reports were removed. Drug names were standardized using the RxNorm drug normalization naming system to standardize the drug names in the FAERS database. The international medical terminology dictionary version 27.1 (MedDRA 27.1) was used to match the primary terms (PT) for adverse events related to “bone density reduction and osteoporosis”.

After standardizing the drug names and adverse event names, the adverse event reports for the primary suspected (PS) drugs related to bone density reduction and osteoporosis were collected. These adverse event reports were characterized by gender, age, weight, indications, reporting country, and outcomes.

### 2.2 Signal analysis algorithm

**FIGURE 1:**
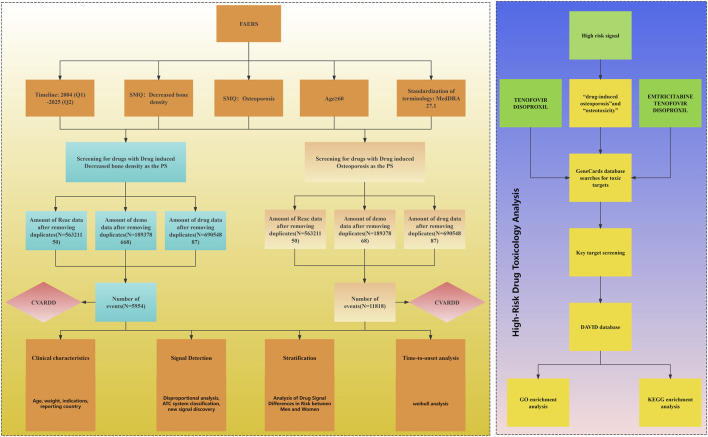
Disproportionality analysis process for drug-related bone density reduction and osteoporosis adverse events. Abbreviations: Faers, fda adverse event reporting system; SMQ, standardised MedDRA queries; PS, primary suspected; CVARDD, Canada vigilance adverse reaction online database; GO, gene ontology; KEGG, kyoto encyclopedia of genes and genomes.

In this study, the disproportionality analysis (DPA) algorithm, commonly used in pharmacovigilance studies, was employed to detect potential signals for adverse events related to bone density reduction and osteoporosis. The disproportionality analysis algorithm is a widely used data mining method that analyzes the correlation between drugs and adverse reactions by comparing the observed frequency ratios in exposed and unexposed populations using a 2 × 2 contingency table, as shown in Supplementary Table S1. In this study, the Reporting Odds Ratio (ROR) was used to calculate the signal strength (Rothman et al., 2004). A risk signal was considered present when the lower limit of the 95% confidence interval (CI) of the ROR was greater than 1 and the frequency (a) was greater than 3. To control the risk of false discoveries in multiple comparisons, the Benjamini–Hochberg method was applied using the “p.adjust” function in the “stats” package of R. A two-tailed test was performed, and statistical significance was set at a false discovery rate (FDR) adjusted p-value (FDR p) below 0.05 (Jing et al., 2022). Effective signals were determined as new adverse event signals when they were not mentioned in the FDA drug label (https://www.accessdata.fda.gov/scripts/cder/daf/index.cfm). All data in this study were processed and analyzed using R4.4.0 and MS Excel software. The data extraction and analysis process is illustrated in Figure 1.

### 2.3 Toxicological analysis

To further investigate the mechanisms of high-risk drugs associated with bone density reduction and osteoporosis, we conducted a toxicological analysis of Tenofovir Disoproxil and Emtricitabine Tenofovir Disoproxil. First, we extracted known target information for these two drugs from the PubChem and SwissTarget Prediction databases, and combined it with data from the GeneCards database to filter out toxicity targets related to “drug-induced osteoporosis” and “osteotoxicity.”

Using a Venn diagram analysis, we identified the intersection of toxicity targets associated with these drugs and osteoporosis, revealing their potential mechanisms of action. After target screening, we performed Gene Ontology (GO) analysis and KEGG pathway enrichment analysis of the intersecting targets using the DAVID database to explore their potential biological processes.

## 3 Results

### 3.1 Population characteristics of drug-related bone density reduction and osteoporosis adverse events

**FIGURE 2:**
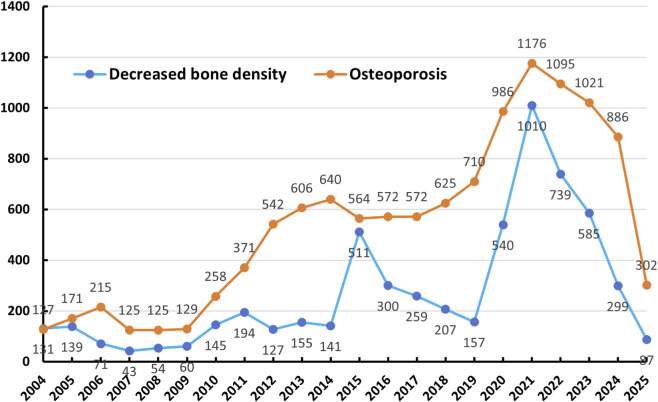
Trend of adverse reaction reporting between drug related bone density reduction and osteoporosis.

As of Q2 2025, the FAERS database reported 5,954 adverse events related to decreased bone density. As shown in Figure 2, 11,818 adverse events related to osteoporosis were recorded. From 2004 to 2025, the number of adverse event reports generally showed a fluctuating trend rather than a linear increase proportional to the database size.

Notably, a disproportionate surge was observed between 2020 and 2021, where reports of 'Decreased bone density’ increased by 87% (from 540 to 1,010 cases) and 'Osteoporosis’ peaked at 1,176 cases.

Subsequently, a distinct downward trend occurred from 2022 to 2024, diverging from the continued expansion of the FAERS database. This 'peak-and-fall’ pattern suggests that the reported events were driven by specific pharmacovigilance signals or clinical factors during the peak period, rather than being an artifact of overall database growth.

Table 1 shows the characteristics of the populations for bone density reduction and osteoporosis adverse events in the elderly. Among the reports for decreased bone density, 65.1% were female, 34.0% were male, and 0.8% had unknown gender. Regarding age distribution, the group aged 65–85 years (54.3%) accounted for the largest proportion of known-age reports. Weight was primarily concentrated in the 50–100 kg range (17.4%), but 78.8% of the population had unknown weight. Notably, HIV infection (36.9%) was the most common indication, followed by osteoporosis (20.8%) and postmenopausal osteoporosis (5.0%). The countries with the highest number of reports were the United States (70.0%), Canada (5.5%), and China (1.6%). For osteoporosis, 76.5% of the reports were from females, 22.7% from males, and 0.8% had unknown gender. In terms of age, the group aged 18–65 years (59.4%) accounted for the largest proportion of known-age reports. Weight was primarily concentrated in the 50–100 kg range (29.9%), but 63.9% of the population had unknown weight. Notably, rheumatoid arthritis (13.6%) was the most common indication, followed by HIV infection (11.8%) and osteoporosis (5.3%). The countries with the highest number of reports were the United States (56.2%), Canada (12.3%), and Germany (2.1%).

**TABLE 1:** Population characteristics of adverse events related to drug-induced Decreased bone density and osteoporosis.

Characteristics	Decreased bone density		Osteoporosis	
Number of events	5954		11,818	
Gender, number (%)				
Female	3878 (65.1%)		9039 (76.5%)	
Male	2027 (34.0%)		2686 (22.7%)	
Miss	49 (0.8%)		93 (0.8%)	
Age, number (%)				
Median age	67		70	
60–85	5778 (97.10%)		11,285 (95.5%)	
>85	176 (3.0%)		533 (4.5%)	
Weight (KG), number (%)				
<50	151 (2.50%)		473 (4.0%)	
50∼100	1035 (17.40%)		3532 (29.9%)	
>100	77 (1.30%)		267 (2.3%)	
Miss	4692 (78.80%)		7546 (63.9%)	
Top 5 indication, number(%)	HIV INFECTION	2201 (36.9%)	RHEUMATOID ARTHRITIS	1614 (1614,13.6%)
	OSTEOPOROSIS	1239 (20.8%)	HIV INFECTION	1404 (11.8%)
	OSTEOPOROSIS POSTMEHOPAUSAL	303 (5.0%)	OSTEOPOROSIS	627 (5.3%)
	RHEUMATOID ARTHRITIS	192 (3.2%)	GASTROOESOPHAGEAL REFLUX DISEASE (420)	420 (3.5%)
	OSTEOPENIA	81 (1.3%)	MULTIPLE SCLEROSIS	234 (1.9%)
Top 5 Reported Countries, number (%)	United States	4170 (70.0%)	United States	6666 (56.2%)
	Canada	328 (5.5%)	Canada	1459 (12.3%)
	CHINA	97 (1.6%)	GERMANY	256 (2.1%)
	JAPAN	86 (1.4%)	BRAZIL	253 (2.1%)
	GREAT BRITAIN	36 (0.6%)	GREAT BRITAIN	215 (1.8%)

### 3.2 Risk drug analysis in the elderly population

**FIGURE 3:**
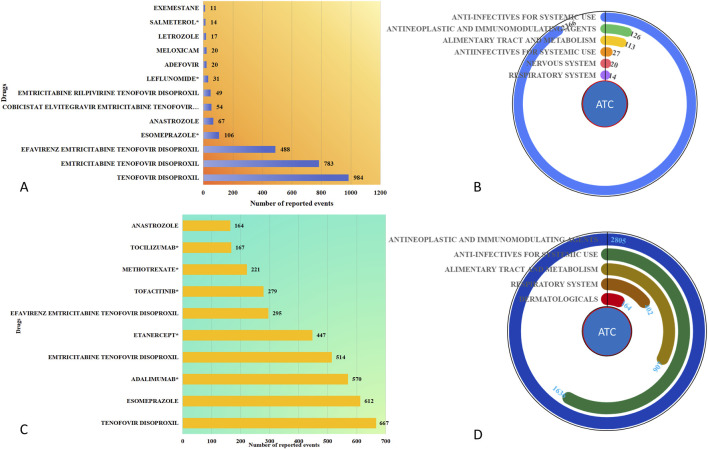
Analysis of Drug-Related Bone Density Reduction and Osteoporosis Risk Signals in the General Population. **(A)** Bar chart of top risk signal drugs for bone density reduction; **(B)** Donut chart of ATC classifications for bone density reduction risk signals; **(C)** Bar chart of top risk signal drugs for osteoporosis; **(D)** Donut chart of ATC classifications for osteoporosis risk signals.

There are 18 drugs associated with adverse events related to drug-induced bone density reduction, as shown in Figure 3A. The top five drugs are Tenofovir Disoproxil (N = 984), Emtricitabine Tenofovir Disoproxil (N = 783), Efavirenz Emtricitabine Tenofovir Disoproxil (N = 488), Esomeprazole (N = 106), and Anastrozole (N = 67). As detailed in Table 2, the top five drugs associated with decreased bone density, ranked by the number of reports, all exhibited highly robust risk signals. These include Tenofovir Disoproxil (N = 984, ROR = 665.79, 95% CI: 611.91–724.42), Emtricitabine Tenofovir Disoproxil (N = 783, ROR = 713.48, 95% CI: 648.66–784.79), Efavirenz Emtricitabine Tenofovir Disoproxil (N = 488, ROR = 691.12, 95% CI: 613.48–778.59), Esomeprazole (N = 106, ROR = 3.98, 95% CI: 3.28–4.82), and Anastrozole (N = 67, ROR = 8.44, 95% CI: 6.63–10.75). The drugs were further classified according to the ATC classification system, as shown in Figure 3B, where Anti-infectives for Systemic Use (N = 2,366) was the most prevalent drug category, followed by Antineoplastic and Immunomodulating Agents (N = 126).

**TABLE 2:** Disproportionality analysis of top 10 drugs associated with decreased bone density and osteoporosis in older adults reported to the FAERS database (2004–2025 Q2).

Adverse event	Drug	a	ROR (95% CI)	p value	p adjust
Decreased bone density	TENOFOVIR DISOPROXIL	984	665.79 (611.91–724.42)	0	0
	EMTRICITABINE TENOFOVIR DISOPROXIL	783	713.48 (648.66–784.79)	0	0
	EFAVIRENZ EMTRICITABINE TENOFOVIR DISOPROXIL	488	691.12 (613.48–778.59)	0	0
	ESOMEPRAZOLE *	106	3.98 (3.28–4.82)	1.48E-51	4.41E-49
	ANASTROZOLE *	67	8.44 (6.63–10.75)	4.68E-94	1.39E-91
	COBICISTAT ELVITEGRAVIR EMTRICITABINE TENOFOVIR DISOPROXIL	54	348.17 (254.01–477.24)	1.19E-111	3.54E-109
	EMTRICITABINE RILPIVIRINE TENOFOVIR DISOPROXIL	49	302.61 (218.67–418.77)	6.02E-99	1.80E-96
	LEFLUNOMIDE *	31	6.00 (4.21–8.55)	1.67E-28	4.97E-26
	ADEFOVIR	20	40.41 (25.80–63.30)	3.23E-25	9.62E-23
	MELOXICAM *	20	10.24 (6.58–15.92)	4.77E-14	1.42E-11
Osteoporosis	TENOFOVIR DISOPROXIL	667	167.20 (152.77–182.98)	0	0
	ESOMEPRAZOLE *	612	12.23 (11.27–13.28)	0	0
	ADALIMUMAB *	570	2.16 (1.99–2.35)	2.64E-75	1.76E-72
	EMTRICITABINE TENOFOVIR DISOPROXIL	514	172.62 (155.75–191.32)	0	0
	ETANERCEPT *	447	1.31 (1.19–1.44)	2.28E-08	1.53E-05
	EFAVIRENZ EMTRICITABINE TENOFOVIR DISOPROXIL	295	154.34 (135.09–176.33)	0	0
	TOFACITINIB *	279	1.90 (1.69–2.14)	6.61E-27	4.42E-24
	METHOTREXATE *	221	2.12 (1.85–2.42)	2.72E-29	1.82E-26
	TOCILIZUMAB *	167	4.51 (3.87–5.26)	3.81E-98	2.55E-95
	ANASTROZOLE *	164	10.57 (9.05–12.35)	8.88E-298	5.94E-295

a = number of reports; ROR, reporting odds ratio; CI, confidence interval.

Asterisks (*) denote new disproportionality signals not previously documented in the respective drug labeling.

Further comparison with the drug labels revealed that eight drugs, including Esomeprazole (N = 106, ROR = 3.98), Leflunomide (N = 31, ROR = 6), Salmeterol (N = 14, ROR = 1.94), Lamivudine (N = 7, ROR = 6.18), and Lopinavir Ritonavir (N = 5, ROR = 6.33), did not directly mention bone density reduction as an adverse event in the product labeling. These newly identified adverse event signals are worth further clinical attention, as detailed in Supplementary Table S2.

For osteoporosis-related adverse events, 63 drugs were identified as risk drugs, as shown in Figure 3C. The top five drugs are Tenofovir Disoproxil (N = 667), Esomeprazole (N = 612), Adalimumab (N = 570), Emtricitabine Tenofovir Disoproxil (N = 514), and Etanercept (N = 447). For osteoporosis-related adverse events, the top five drugs ranked by report count (Table 2) also demonstrated significant associations. The leading drugs were Tenofovir Disoproxil (N = 667, ROR = 167.20, 95% CI: 152.77–182.98), followed by Esomeprazole (N = 612, ROR = 12.23, 95% CI: 11.27–13.28), Adalimumab (N = 570, ROR = 2.16, 95% CI: 1.99–2.35), Emtricitabine Tenofovir Disoproxil (N = 514, ROR = 172.62, 95% CI: 155.75–191.32), and Etanercept (N = 447, ROR = 1.31, 95% CI: 1.19–1.44). According to the ATC classification system (Figure 3D), the largest drug category was Antineoplastic and Immunomodulating Agents (N = 2,805). Notably, after correcting for terminology variations, 'Anti-infectives for systemic use’ emerged as the second-largest category with 1,636 reports. This was followed by Alimentary Tract and Metabolism (N = 907). The high prevalence of anti-infectives is consistent with the significant signals observed for antiretroviral drugs in our specific drug analysis.

Further comparison with the drug labels revealed that 32 drugs, including Adalimumab (N = 570, ROR = 2.16), Etanercept (N = 447, ROR = 1.31), Tofacitinib (N = 279, ROR = 1.9), Methotrexate (N = 221, ROR = 2.12), and Tocilizumab (N = 167, ROR = 4.51), did not directly mention osteoporosis as an adverse event in the product labeling. These newly identified adverse event signals are also worth further clinical attention, as detailed in Supplementary Table S2.

### 3.3 Gender differences in risk drugs for bone density reduction and osteoporosis

**FIGURE 4:**
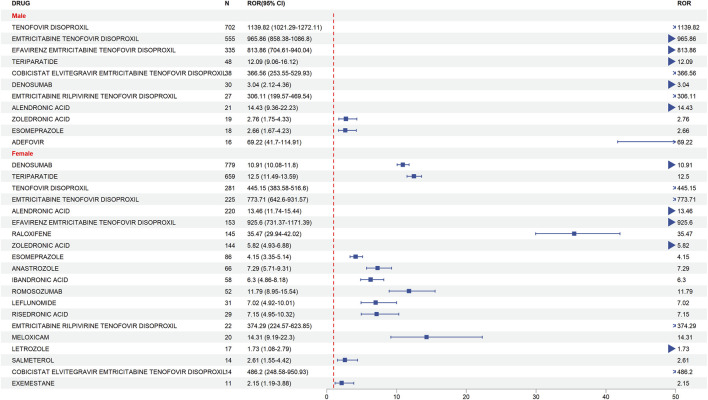
Forest Plot of gender differences in risk drugs for bone density reduction.

To elucidate gender-specific susceptibilities to drug-induced bone toxicity, we performed a stratified disproportionality analysis. Regarding bone density reduction (Figure 4), a stark dichotomy in drug risk profiles between genders emerged. Male patients exhibited overwhelmingly dominant risk signals associated almost exclusively with antiretroviral therapies. Notably, Tenofovir Disoproxil demonstrated an extreme association with bone density reduction in men (N = 702, ROR = 1139.82, 95% CI: 1021.29–1272.11), which was substantially higher than the signal observed in women (N = 281, ROR = 445.15, 95% CI: 383.58–516.60). Conversely, while female patients also demonstrated significant risks from antiviral agents, they uniquely presented a broader risk spectrum driven by bone metabolic regulators, such as Denosumab (N = 779, ROR = 10.91, 95% CI: 10.08–11.80) and Teriparatide (N = 659, ROR = 12.50, 95% CI: 11.49–13.59).

Heatmap analysis of the shared risk signals (Figure 6A) further illustrates these nuances: while Tenofovir Disoproxil and Emtricitabine Tenofovir Disoproxil (male ROR = 965.86, 95% CI: 858.38–1086.80) had profoundly higher toxicities in men, complex fixed-dose combinations like Efavirenz/Emtricitabine/Tenofovir Disoproxil (female ROR = 925.60, 95% CI: 731.37–1171.39) and Cobicistat/Elvitegravir/Emtricitabine/Tenofovir Disoproxil (female ROR = 486.20, 95% CI: 248.58–950.93) exhibited stronger signal intensities in women, indicating that gender plays a critical modulating role even within the same antiretroviral drug class.

**FIGURE 5:**
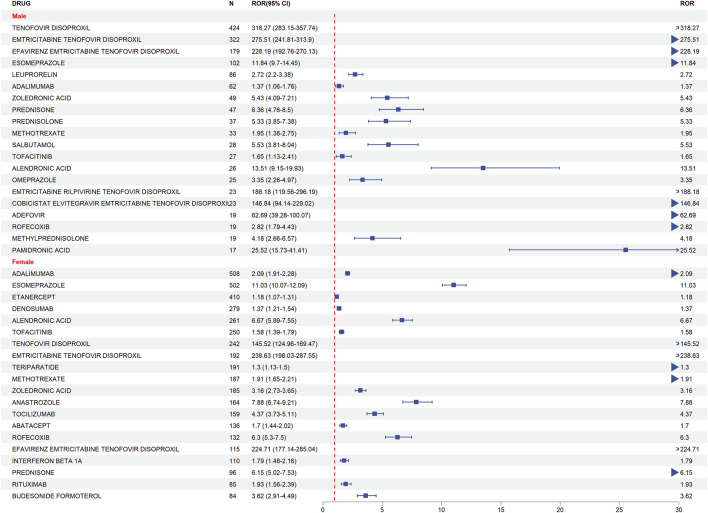
Forest Plot of gender differences in risk drugs for osteoporosis.

This gender-dependent risk pattern was mirrored in osteoporosis events (Figure 5). Tenofovir Disoproxil again showed a disproportionately higher risk in men (N = 424, ROR = 318.27, 95% CI: 283.15–357.74) compared to women (N = 242, ROR = 145.52, 95% CI: 124.96–169.47). However, the osteoporosis risk spectrum in female patients was distinctly expanded to include strong signals from proton pump inhibitors and immunomodulators, driven primarily by Esomeprazole (N = 502, ROR = 11.03, 95% CI: 10.07–12.09) and Adalimumab (N = 508, ROR = 2.09, 95% CI: 1.91–2.28).

**FIGURE 6:**
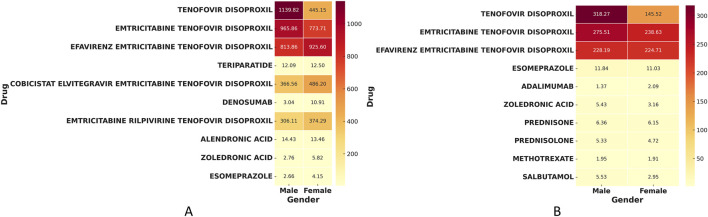
Heatmap of shared risk signals for bone density reduction and osteoporosis in both men and Women. **(A)** Heatmap of ROR values for shared risk signals for bone density Reduction. **(B)** Heatmap of ROR values for shared risk signals for osteoporosis.

The corresponding heatmap for shared osteoporosis signals (Figure 6B) visually reinforces this dichotomy. It highlights that fundamental antiretrovirals like Tenofovir Disoproxil and Emtricitabine Tenofovir Disoproxil (male ROR = 275.51, 95% CI: 241.81–313.90) remain heavily male-predominant risks. In contrast, female vulnerabilities are elevated across certain combination therapies (e.g., Emtricitabine/Tenofovir Disoproxil: female ROR = 238.63, 95% CI: 198.03–287.55; Efavirenz/Emtricitabine/Tenofovir Disoproxil: female ROR = 224.71, 95% CI: 177.14–285.04) alongside a wider array of anti-inflammatory and metabolic medications.

### 3.4 Toxicological analysis

Tenofovir Disoproxil and Emtricitabine Tenofovir Disoproxil are the drugs with the highest number of reported events and the highest ROR values, with both being used for the treatment of HIV. To further explore their potential risks in osteoporosis, we conducted a toxicological analysis. Target information for these drugs was retrieved from the PubChem and SwissTarget Prediction databases, while toxicity targets related to “drug-induced osteoporosis” and “osteotoxicity” were obtained from the GeneCards database.

**FIGURE 7:**
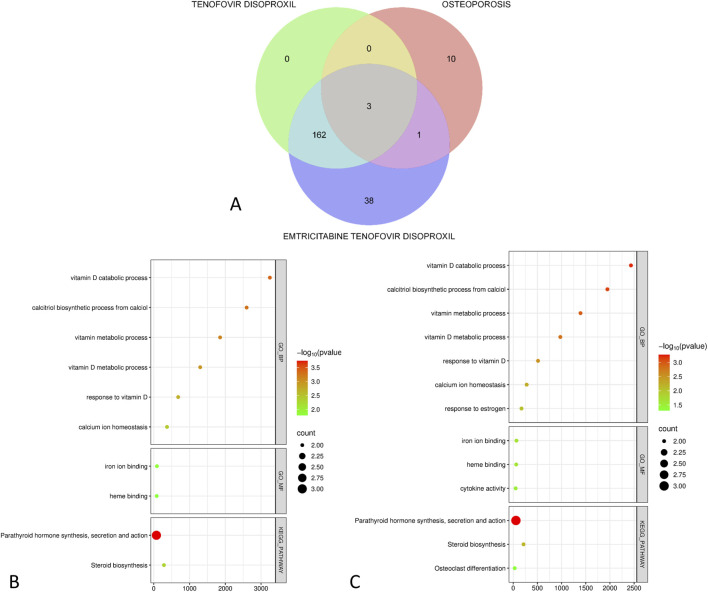
Toxicological analysis of high-risk signal drugs. **(A)** Venn diagram of the intersecting toxicity targets between high-risk drugs and osteoporosis; **(B)** GO and KEGG pathway enrichment analysis of the intersecting targets for Tenofovir Disoproxil; **(C)** GO and KEGG pathway enrichment analysis of the intersecting targets for Emtricitabine Tenofovir Disoproxil.

As shown in the Venn diagram in Figure 7A, Tenofovir Disoproxil has three intersecting toxicity targets with osteoporosis, while Emtricitabine Tenofovir Disoproxil has four intersecting targets. Further Gene Ontology (GO) and KEGG enrichment analyses were performed using the DAVID database.

Figure 7B shows that the intersecting targets of Tenofovir Disoproxil are highly focused on key endocrine regulatory axes in bone metabolism. The GO_BP (biological process) enrichment primarily highlights processes related to vitamin D/calcitriol metabolism and response (e.g., vitamin D metabolic/catabolic process, calcitriol biosynthetic process, response to vitamin D), further pointing to calcium ion homeostasis maintenance. At the KEGG pathway level, significant enrichment was found in the synthesis, secretion, and action pathways of parathyroid hormone (PTH), along with associations to steroid biosynthesis. These results suggest that Tenofovir Disoproxil may disrupt the “vitamin D—calcium homeostasis—PTH” regulatory network, leading to metabolic imbalance in bone mass and increasing the risk of osteoporosis.

Figure 7C shows that the intersecting targets of Emtricitabine Tenofovir Disoproxil also focus on endocrine regulation of bone metabolism. The GO_BP enrichment is significantly related to vitamin D/calcitriol metabolism and response, calcium ion homeostasis, and estrogen response, indicating that its potential effects may extend to sex hormone regulation. In addition to significant enrichment in the PTH pathway and steroid biosynthesis, the KEGG pathways also associate with osteoclast differentiation. Overall, this combination drug may disrupt the “vitamin D—calcium homeostasis—PTH/sex hormones” regulatory network and influence osteoclast-related bone remodeling processes, thus promoting bone homeostasis imbalance and increasing the risk of osteoporosis.

### 3.5 External validation with the canadian database

To further ensure the accuracy of the study results, we analyzed adverse event reports related to bone density reduction and osteoporosis in the Canadian CVARDD database. Among the reports related to bone density reduction, Esomeprazole, Leflunomide, and Meloxicam exhibited risk signals for bone density reduction in both databases. Regarding osteoporosis-related adverse events, 33 common risk signals for osteoporosis were reported consistently in both the FAERS and CVARDD databases, which further validates the robustness of the results.

**FIGURE 8:**
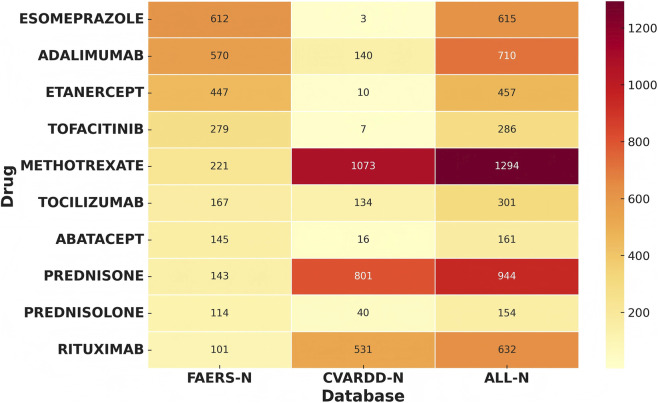
Heatmap of risk drugs from FAERS, CVARDD, and combined data sources.

As shown in Figure 8, Esomeprazole (FAERS = 612, CVARDD = 3) shows a relatively low risk in both databases, indicating consistent adverse event risk in both datasets. Adalimumab (FAERS = 570, CVARDD = 140) exhibited a high risk in both databases, suggesting that the risk distribution for this drug is consistent across databases, further supporting the reliability of the study findings. Etanercept (FAERS = 447, CVARDD = 10) showed a higher risk in the FAERS database but a lower risk in the CVARDD database, which could be attributed to differences in data sources or geographic factors. Tofacitinib (FAERS = 279, CVARDD = 7) displayed moderate risk in both databases, further enhancing the consistency and robustness of the results. Methotrexate (FAERS = 221, CVARDD = 1073) showed a high risk in both databases, with the CVARDD database indicating an even more prominent risk, suggesting that the impact of this drug on bone density has been validated across both databases.

These results indicate that the findings from the FAERS and CVARDD databases mutually support each other, enhancing the reliability and accuracy of the study conclusions. Additionally, this suggests that the reporting of adverse drug reactions in different databases may be influenced by geographic or sample differences.

### 3.6 Induction time

The analysis of induction time for drug adverse reactions is of significant importance for drug safety monitoring, clinical medication guidance, regulatory decision-making, and drug development improvement. Due to the high rate of missing data for induction time in bone density reduction, we focused on analyzing the top 5 risk drugs for osteoporosis adverse events that were reported in both FAERS and CVARDD.

Weber distribution curve parameters are used for this analysis: when the shape parameter (β) is less than 1, with its 95% confidence interval (CI) less than 1, the adverse event (AE) incidence is considered to decrease over time (early failure-type curve). When β equals or is close to 1, and its 95% CI includes 1, the AE incidence continues to occur over time (random failure-type curve). When β is greater than 1, with its 95% CI not including 1, the AE incidence is considered to increase over time (wear-out failure-type curve).

As shown in Table 3, the results reveal significant differences in the induction time characteristics of the drugs. Tenofovir Disoproxil and its combination formulations have a longer time-to-onset (TTO) and exhibit a “wear-out failure-type curve” characteristic. The median induction time for Tenofovir Disoproxil was 1,272 days (β = 1.37, 95% CI: 1.22–1.52), indicating that as the medication duration increases, the adverse event incidence gradually rises. The findings were consistent in both male (β = 1.44) and female (β = 1.29) subgroups, suggesting that the risk of osteoporosis is closely related to cumulative medication duration. Similarly, Emtricitabine Tenofovir Disoproxil and its gender subgroups also had β values greater than 1 (1.30–1.49), showing a wear-out trend, which suggests that long-term medication use may lead to ongoing damage to bone structure.

**TABLE 3:** Weibull distribution parameters for time-to-onset of drug-related osteoporosis.

Drug	TTO (days)		Weibull distribution		
	Case reports	Median (day)	Scale parameter: α(95%CI)	Shape parameter: β(95%CI)	Type
TENOFOVIR DISOPROXIL	226	1272	1463.98 (1318.36∼1609.60)	1.37 (1.22∼1.52)	Wear-out failure curve
ESOMEPRAZOLE	44	1374	1497.14 (1256.71∼1737.58)	1.95 (1.48∼2.43)	Wear-out failure curve
ADALIMUMAB	125	354	625.48 (482.75∼768.21)	0.81 (0.70∼0.93)	Early failure
EMTRICITABINE TENOFOVIR DISOPROXIL	177	1403.5	1522.51 (1355.64∼1689.39)	1.41 (1.23∼1.58)	Wear-out failure curve
ETANERCEPT	46	626	851.19 (524.77∼1177.61)	0.80 (0.60∼1.00)	Early failure
TENOFOVIR DISOPROXIL(M)	133	1248.5	1468.48 (1302.05∼1670.92)	1.44 (1.23∼1.64)	Wear-out failure curve
TENOFOVIR DISOPROXIL(F)	93	1435	1469.99 (1230.72∼1709.25)	1.29 (1.07∼1.51)	Wear-out failure curve
EMTRICITABINE TENOFOVIR DISOPROXIL(M)	104	1386	1578.37 (1365.05∼1791.68)	1.49 (1.25∼1.74)	Wear-out failure curve
EMTRICITABINE TENOFOVIR DISOPROXIL(F)	73	1449	1517.81 (1242.37∼1787.26)	1.30 (1.06∼1.56)	Wear-out failure curve

Abbreviation: TTO, time-to-onset; CI, confidence interval.

Esomeprazole also showed significant wear-out failure-type characteristics. The median induction time was 1,374 days (β = 1.95, 95% CI: 1.48–2.43), indicating that the risk of osteoporosis significantly increases with prolonged use. This may be related to the long-term effects of acid suppression therapy on calcium absorption and bone metabolism disruption.

Adalimumab and Etanercept exhibited an “early failure-type curve.” The median induction time for Adalimumab was 354 days (β = 0.81, 95% CI: 0.70–0.93), and for Etanercept, it was 626 days (β = 0.80, 95% CI: 0.60–1.00), both suggesting that adverse events occur more frequently in the early stages of medication. This may be due to the early effects of immunomodulatory drugs on bone metabolic balance.

**FIGURE 9:**
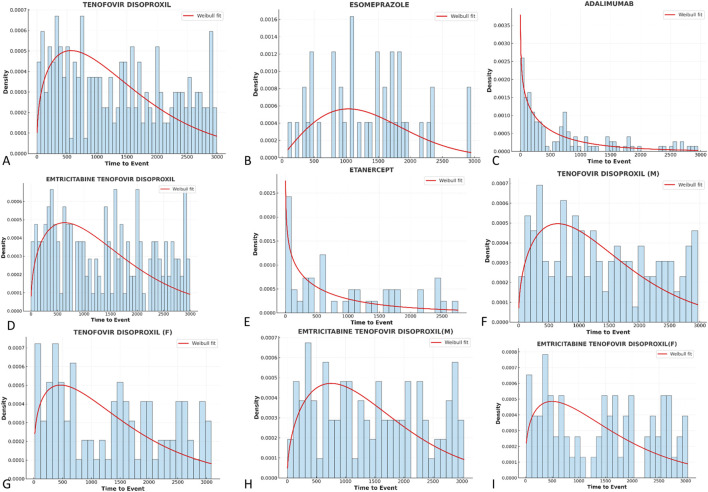
Weber analysis of induction time for osteoporosis high-risk signal Drugs. **(A)** Tenofovir disoproxil induction time weber distribution curve **(B)** Esomeprazole induction time weber distribution curve **(C)** Adalimumab induction time weber distribution curve **(D)** Emtricitabine tenofovir disoproxil induction time weber distribution curve **(E)** Etanercept induction time weber distribution curve **(F)** Tenofovir disoproxil (male) induction time weber distribution curve **(G)** Tenofovir disoproxil (female) induction time weber distribution curve **(H)** Emtricitabine tenofovir disoproxil (male) induction time weber distribution curve **(I)** Emtricitabine tenofovir disoproxil (female) induction time weber distribution curve.

Overall, Tenofovir-class drugs are most prominently associated with wear-out risks related to long-term use, while Adalimumab and Etanercept may induce osteoporosis risk early in treatment. The Weber distribution curves further confirm this, with early failure-type curves appearing in Figures 9C,E, while wear-out failure-type curves are shown in Figures 9A,B,D,F–I.

These results indicate that different drugs exhibit significant variations in the time distribution patterns of osteoporosis-related adverse reactions, suggesting that clinical monitoring and management of bone metabolism risks should be strengthened during long-term medication use.

## 4 Discussion

Decreased bone density and osteoporosis are potential serious bone metabolism-related adverse drug reactions (ADRs) that can significantly affect the physiological function and quality of life of elderly patients, potentially leading to fractures and permanent damage (Rizzoli et al., 2011). With the widespread use of antiviral drugs, immunomodulatory drugs, and proton pump inhibitors, the associated bone metabolic risks have gradually gained attention. However, there is still limited systematic research on drug-related bone density reduction and osteoporosis in the elderly population, especially lacking multi-drug comparisons and gender stratification analysis based on real-world data.

This study, based on FAERS database data from 2004 to 2025, used disproportionality analysis (DPA) methods to systematically identify potential high-risk drugs, and its robustness was validated through the CVARDD database (Gibbons et al., 2025). Furthermore, the induction time modeling was combined to reveal the temporal evolution patterns of bone metabolism adverse reactions, providing real-world evidence for medication risk management in elderly patients.

### 4.1 Characteristics of vulnerable populations and clinical focus

Population characteristic analysis shows that women make up a significantly higher proportion of reports for adverse events related to bone density reduction (65.1%) and osteoporosis (76.5%), suggesting that women may be the primary susceptible group for drug-related bone metabolic damage. This difference may arise from multiple mechanisms: First, the decline in estrogen levels leads to decreased osteoblastic activity and increased osteoclastic activity, making women more prone to bone loss under pharmacological interventions (Eastell et al., 2019). Second, women have a higher prevalence of chronic diseases like osteoporosis and rheumatoid arthritis, which increases exposure to related adverse drug reactions due to long-term use of anti-inflammatory or immunomodulatory drugs (Buttgereit et al., 2024). Additionally, older women experience age-related declines in calcium absorption, vitamin D metabolism, and renal function reserves, which may further exacerbate the adverse effects of drugs on bone metabolism.

Age distribution results indicate that the 65–85 age group accounts for the largest proportion of adverse events in both categories, suggesting that older age is a high-risk period for drug-induced bone metabolic abnormalities. With aging, slower bone remodeling, degradation of trabecular microstructure, and polypharmacy are common, all of which may amplify the negative impact of drugs on bone density (Liu et al., 2025). Although the 50–100 kg weight group appeared most prevalent among reported cases, data on patient weight were missing in a significant proportion of reports (>60%). Given this high rate of missing data, we refrained from drawing definitive conclusions regarding dose-to-weight ratios or pharmacokinetic variations. This limitation highlights the critical need for more complete demographic recording in future real-world monitoring to facilitate precise risk assessment.

Indication analysis revealed that HIV infection accounted for the highest proportion of reports related to bone density reduction (36.9%), while rheumatoid arthritis (13.6%) and HIV infection (11.8%) were the leading indications for osteoporosis. This suggests that the immunosuppressive state and long-term antiviral therapy may jointly impact bone metabolic pathways, increasing the risk of drug-induced bone damage (Biver, 2022). Particularly, patients on antiretroviral therapy (ART) may experience chronic inflammation, tubular toxicity, and vitamin D metabolism abnormalities, which further exacerbate bone loss (Finnerty et al., 2017). The majority of reports came from the United States and Canada, reflecting the robustness of drug safety monitoring systems in North America, and highlighting the need for further global sharing and standardization of drug safety data.

In summary, elderly women, long-term users of antiviral or immunomodulatory drugs, and individuals over 65 years of age are key targets for the prevention and control of bone metabolic-related adverse reactions. Clinicians should enhance bone density monitoring and nutritional metabolic assessments during the early stages of treatment.

### 4.2 Gender differences and risk drug spectrum

This study revealed significant gender differences in drug-related bone density reduction and osteoporosis. In bone density reduction events, antiviral drugs, especially Tenofovir Disoproxil (ROR = 1139.82) and its combination formulation Emtricitabine Tenofovir Disoproxil (ROR = 965.86), were the high-risk drugs for men, whereas women exhibited higher risks with bone metabolic regulators such as Denosumab (ROR = 10.91) and Teriparatide (ROR = 12.5). These differences not only reflect medication background variations but also illustrate the different mechanisms of action of drugs in gender-specific physiological and bone metabolic pathways.

In men, Tenofovir Disoproxil and its combination formulations induce mitochondrial dysfunction and renal proximal tubule damage by inhibiting mitochondrial DNA polymerase-γ (DNA polymerase-γ), resulting in phosphate loss, Fanconi syndrome, and hypophosphatemia, which ultimately lead to impaired bone mineralization and demineralization. Furthermore, Tenofovir downregulates osteoblast key transcription factors such as RUNX2 and osteocalcin, inhibiting bone formation, while enhancing osteoclast activity, thereby molecularly exacerbating bone density reduction (Mazon et al., 2018; Kimura et al., 2024). Many male HIV patients require long-term combination antiretroviral therapy (ART), and the cumulative effects of these drugs, along with kidney-bone axis damage mechanisms, may explain the high ROR values observed (Tomazic et al., 2007).

In women, Denosumab, a monoclonal RANKL antibody, effectively inhibits osteoclastogenesis by blocking the RANKL/RANK signaling pathway. However, after discontinuation, the rebound effect of RANKL leads to a significant increase in bone resorption in the short term, resulting in rapid bone loss and increased fracture risk (Diab and Watts, 2014). Teriparatide (recombinant human parathyroid hormone 1–34) stimulates bone formation intermittently, but when used long-term or at inappropriate doses in elderly women with declining metabolic reserves, it may activate the RANKL pathway and cause excessive bone remodeling, leading to bone structure degeneration and decreased bone density under a state of bone metabolic imbalance (Blick et al., 2009). Additionally, estrogen deficiency downregulates estrogen receptor-α (ERα) signaling in female osteocytes, reducing the tolerance to drug-induced metabolic stress, thereby amplifying adverse drug effects on bone metabolism (Cui et al., 2024).

In osteoporosis-related events, male risk drugs were also primarily antiviral drugs (Tenofovir Disoproxil, ROR = 318.27; Emtricitabine Tenofovir Disoproxil, ROR = 275.51), while women’s risk drugs were represented by immunomodulatory drugs and proton pump inhibitors (PPIs) (Adalimumab, ROR = 2.09; Esomeprazole, ROR = 11.03). In men, long-term Tenofovir exposure may induce osteoporosis through energy metabolism disorders in bone cells and mechanisms of renal bone disease. The damage mechanisms include: ① impaired tubular reabsorption of phosphate; ② reduced synthesis of 1,25-dihydroxyvitamin D; ③ elevated serum alkaline phosphatase, leading to impaired bone matrix calcification (Kim et al., 2023).

Women taking PPIs show a significantly increased fracture risk. The molecular mechanisms include: decreased calcium solubility and impaired active absorption due to suppressed gastric acid secretion, downregulation of the calcium transporter TRPV6 in the intestine under long-term low-acid conditions (Moledina and Perazella, 2016); compensatory increases in parathyroid hormone (PTH) promoting bone resorption; and interference with the local microenvironment of bone remodeling via suppression of gastrin-related signaling (Huang et al., 2024). Additionally, Esomeprazole may interfere with the Wnt/β-catenin signaling pathway, inhibiting osteoblast differentiation, thereby further impairing bone formation (Luk et al., 2013). For immunomodulatory agents like Adalimumab (anti-TNF-α monoclonal antibody), while improving arthritis symptoms, it can suppress TNF-α-mediated osteoblast activity recovery and bone remodeling, which may lead to bone metabolic imbalance with long-term use (Chan et al., 2015).

Gender differences are not only reflected in the types of drugs but also in the sensitivity of molecular pathways to responses. Male bone metabolism is primarily regulated by the androgen-IGF-1 axis, where a decrease in androgen levels reduces bone formation rate. In contrast, women’s bone metabolism is regulated by the estrogen-RANKL/OPG axis, where a decline in estrogen levels significantly enhances osteoclastic activity. This difference results in varying effects of the same drug on bone homeostasis through different hormone-dependent pathways, causing differences in ROR intensity (Ga et al., 2000). In conclusion, drug-induced bone metabolic damage exhibits distinct gender specificity, and the mechanisms involve endocrine regulation, mitochondrial energy metabolism, osteocyte signal transduction, and other biological processes. Clinical drug safety monitoring should incorporate gender, physiological, and metabolic status into stratified assessments for personalized risk management.

### 4.3 Exploration of potential risk drugs and mechanisms

This study identified 8 new risk signal drugs associated with bone density reduction and 32 with osteoporosis. These include Esomeprazole (ROR = 3.98), Leflunomide (ROR = 6.00), Salmeterol (ROR = 1.94), Lamivudine (ROR = 6.18), and Lopinavir/Ritonavir (ROR = 6.33). These drugs were not listed in the product labeling as having adverse effects on bone metabolism, indicating potential risks.

From a pharmacological perspective, proton pump inhibitors (PPIs) inhibit gastric acid secretion, reducing intestinal calcium absorption and TRPV6 channel activity, and may interfere with the Wnt/β-catenin signaling pathway, inhibiting osteoblast differentiation. Immunomodulatory drugs like Leflunomide can suppress DHODH activity and downregulate BMP-2/Smad pathways, hindering bone formation and enhancing RANKL-mediated bone resorption (Li et al., 2017). Antiviral drugs like Lamivudine and Lopinavir/Ritonavir inhibit mitochondrial DNA polymerase-γ, causing mitochondrial dysfunction, energy metabolism abnormalities, and renal hypophosphatemia, which leads to bone demineralization (Sikora et al., 2023). Beta-2 adrenergic agonists like Salmeterol can activate the cAMP/PKA pathway, upregulate RANKL expression, and promote osteoclast activation (Xie et al., 2020).

We further conducted toxicological analyses for Tenofovir Disoproxil (most reported) and Emtricitabine Tenofovir Disoproxil (highest ROR). The results showed that although the overlap in osteoporosis-related toxicity targets was small (3 and 4 targets, respectively), the functional enrichment revealed consistent and biologically directed bone metabolic pathways: both drugs significantly enriched processes related to vitamin D/calcitriol metabolism and calcium ion homeostasis maintenance, and at the KEGG pathway level, they focused on PTH synthesis, secretion, and action pathways, along with steroid biosynthesis. Given that PTH is an important endocrine regulator maintaining blood calcium homeostasis, continuous activation of this pathway may lead to RANKL/OPG regulatory imbalance, enhanced osteoclast activity, and shift bone remodeling from dynamic balance to “net bone loss,” thereby increasing osteoporosis risk (De Leon-Oliva et al., 2023). Additionally, the combination drug further correlated with estrogen response and osteoclast differentiation pathways. Estrogen usually regulates the RANKL–OPG axis in osteoblasts/osteocytes (reducing RANKL, increasing OPG) to inhibit osteoclast differentiation and maturation. Disruption of this signaling may weaken physiological inhibition of bone resorption and promote osteoclast dominance (Cheng et al., 2022).

In conclusion, the findings suggest that the osteoporosis risk of HIV-related antiretroviral drugs may not only result from the previously emphasized kidney-bone metabolic axis disruption and phosphate-related bone demineralization but also from sustained disruption of the vitamin D–calcium homeostasis–PTH endocrine regulatory network, along with a cascade of sex hormone regulation imbalances and osteoclast activation. Based on these insights, it is recommended to include vitamin D metabolism markers, calcium-phosphate homeostasis, and PTH-related biochemical parameters in more targeted monitoring frameworks for drug vigilance and clinical risk management, and to use these as key references for future mechanistic validation and risk stratification.

### 4.4 Induction time characteristics and risk management

The time-to-onset (TTO) analysis revealed significant differences in the temporal distribution characteristics of drug-induced bone metabolic adverse reactions. The study found that Tenofovir Disoproxil and its combination formulations (e.g., Emtricitabine Tenofovir Disoproxil) exhibited a typical “wear-out failure curve” (β > 1), with a median TTO exceeding 1,200 days, indicating that the risk of osteoporosis gradually accumulates with prolonged use. This aligns with the mechanisms of renal tubular damage, phosphate loss, and bone demineralization induced by Tenofovir during long-term treatment. Esomeprazole also showed a significant wear-out failure-type curve (β = 1.95), indicating that long-term acid suppression therapy gradually weakens bone remodeling ability, with significant cumulative risk. In contrast, Adalimumab and Etanercept exhibited early failure-type curves with β values < 1 (0.81 and 0.80, respectively), suggesting that adverse reactions occur more frequently early in treatment, potentially related to the early interference of these drugs with bone metabolism balance and immune inflammation (Jamnitski et al., 2012).

These results suggest that in clinical risk management, differentiated strategies should be adopted based on the induction time characteristics of different drugs: for long-term antiviral medications, it is essential to focus on cumulative bone damage risks and recommend regular monitoring of bone density and serum phosphate levels; for immunomodulatory drugs, bone pain, muscle wasting, and other symptoms should be closely monitored in the early stages of treatment and intervened in a timely manner; for elderly women on long-term PPIs or aromatase inhibitors, baseline bone density should be established at the start of treatment, and medication adherence and safety should be evaluated dynamically.

### 4.5 Limitations

This study has several limitations that should be considered. First, the FAERS and CVARDD databases rely on voluntary reporting, which can lead to underreporting or incomplete data. This reporting bias may result in an incomplete representation of the true incidence of drug-related adverse events, particularly when critical information such as gender, age, or weight is missing from reports. Second, while the disproportionality analysis identifies associations between drugs and adverse events, it cannot establish causality. Confounding factors, such as underlying health conditions, co-medications, or genetic predispositions, may contribute to bone metabolism abnormalities but were not fully accounted for in this analysis. Lastly, the study primarily reflects data from the United States and Canada, which may not fully represent global populations. Geographic differences in healthcare systems, drug usage patterns, and genetic diversity could limit the generalizability of the results, highlighting the need for broader, more inclusive studies.

## 5 Conclusion

This study, by analyzing data from the U.S. FDA Adverse Event Reporting System (FAERS), systematically revealed the risk spectrum of drug-related bone density reduction and osteoporosis in the elderly population. The study found that antiviral drugs, immunomodulatory drugs, and proton pump inhibitors are closely related to bone metabolic abnormalities, and that gender plays a significant role in the risk of drug-induced osteoporosis and bone density reduction. Specifically, for HIV patients, antiretroviral drugs such as Tenofovir and its combination formulations were identified as high-risk drugs for osteoporosis.

Through disproportionality analysis (DPA), we identified several potential high-risk drugs and further explored their mechanisms through toxicological analysis. The study results indicate that these drugs may promote the occurrence of osteoporosis by disrupting the vitamin D–calcium homeostasis–PTH regulatory network and sex hormone regulatory mechanisms. Meanwhile, we identified some drugs that were not explicitly listed in the product labeling as causing bone metabolic adverse reactions, providing new monitoring signals for clinical practice.

Additionally, induction time analysis revealed significant differences in the time-to-onset of bone density reduction and osteoporosis-related adverse reactions across different drugs. Specifically, long-term drugs like Tenofovir and its combination formulations exhibited a clear “wear-out failure curve,” suggesting that these drugs progressively increase the risk of osteoporosis over time. In contrast, immunomodulatory drugs tended to induce adverse reactions early in treatment.

In conclusion, this study provides strong evidence for the clinical risk management of drug-related bone metabolic abnormalities, particularly for elderly patients and HIV-infected individuals. The study emphasizes the importance of drug safety monitoring, especially for long-term medications, and suggests that clinicians should strengthen early monitoring and management of bone metabolic adverse reactions, implementing differentiated risk prevention strategies based on the induction time characteristics of the drugs.

## Data Availability

The original contributions presented in the study are included in the article/Supplementary Material, further inquiries can be directed to the corresponding authors.
